# Regulatory effects of C5a on IL-17A, IL-17F, and IL-23

**DOI:** 10.3389/fimmu.2012.00387

**Published:** 2013-01-09

**Authors:** Jamison J. Grailer, Markus Bosmann, Peter A. Ward

**Affiliations:** Department of Pathology, University of Michigan Medical SchoolAnn Arbor, MI, USA

**Keywords:** complement, C5a, IL-17, macrophage, IL-23

## Abstract

The complement anaphylatoxin, C5a, through binding to its receptors (C5aR or C5L2), has important biological properties for recruitment and activation of phagocytes. C5a has been identified as a powerful modulator of Toll-like receptor-induced cytokine and chemokine production by macrophages. Both the complement system and the interleukin (IL)-17 cytokine family protect against extracellular pathogens by enhancing innate immune functions. On the basis of its concentration, C5a can either positively or negatively modulate the production by macrophages of IL-17 family members as well as IL-23 via the phosphatidylinositol 3-kinase/Akt signaling cascade. C5a can also affect the production and maintenance of IL-17-producing T cells. Using C5a, C5aR, or C5L2 deficiency or blockade, IL-17/IL-23 production and/or IL-17-dependent disease progression has been shown to be substantially modified. The contributions of C5a interaction with its receptors in the production of IL-17/IL-23 and promotion of IL-17-dependent immune responses are reviewed.

## INTRODUCTION

The complement system provides an important first line of defense against extracellular pathogens such as bacteria and fungi. It is composed of several membrane-bound and plasma regulators and activators that can interact with a myriad of cell types. The consequences of complement activation vary depending on the physiological context and timing during an immune (innate or adaptive) response. The effects of its products on complement receptors of immune and phagocytic cells are equally diverse. Complement activation exerts a wide range of effects that makes it ideal as both an effector mechanism and coordinator of immune/inflammatory responses. In this review, we summarize recent findings showing that interleukin (IL)-17 and IL-23 are modulated by the complement activation product, C5a.

### COMPLEMENT ACTIVATION AND EFFECTORS

The activation of the complement cascade is requisite for its involvement in immune responses. Complement activation is thought of mainly as occurring in the intravascular space; however, the components are present not only in the circulation, but also in almost every tissue and fluid. Complement can be activated by any of three different pathways (classical, alternative, or lectin), which converge on the cleavage of C3 into C3a and C3b (for a recent review, see Sarma and Ward, 2011). C3a is an anaphylatoxin that affects cellular functions through interactions with its receptor C3aR. C3b acts as an opsonin and also combines with other components to form the C5 convertase that cleaves C5 into C5a and C5b. C5b, in concert with C6–C9 forms the terminal membrane attack complex. C5a is an anaphylatoxin and also has the ability to act as an activator and chemoattractant for phagocytes by binding to its receptors, C5aR and C5L2. C5a can also alter cytokine and chemokine production by macrophages and other cells. Thus, complement activation is an important first line of defense against pathogens; however, complement components have the ability to substantially alter immune responses by modulating the local cytokine milieu.

### THE IL-17 FAMILY OF CYTOKINES

IL-17 has been reported to contribute to disease severity in several models of autoimmune and chronic inflammatory diseases including multiple sclerosis and collagen-induced arthritis (Nakae et al., 2003; Langrish et al., 2005). Furthermore, IL-17 may be a significant driving force in many acute inflammatory reactions including sepsis (Flierl et al., 2008), ischemia-reperfusion injury (Xue et al., 2011), and acute lung injury (Ferretti et al., 2003). The IL-17 family is comprised of six cytokines (IL-17A–F) that have a similar structure highlighted by four conserved cysteine residues. IL-17 was originally described as being produced primarily by T helper 17 (T_H_17) cells, but innate immune/inflammatory cells have also been shown to release substantial amounts. IL-17 members have an important role in generating and propagating immune responses, including inducing and enhancing cytokine [IL-6, tumor necrosis factor (TNF), IL-1β], and chemokine (IL-8, CCL2) production (Fossiez et al., 1996). The predominant IL-17 family members are IL-17A (herein referred to as IL-17) and IL-17F, which share about 50% sequence identity and are often co-expressed. Although the functions of IL-17 and IL-17F are similar, IL-17 is usually more potent at promoting inflammation, and their effects are not always identical (for a review, see Korn et al., 2009).

The IL-17 family binds to a group of five receptors that have distinct ligand specificities. The best described IL-17 receptor is IL-17R, which binds both IL-17A and IL-17F. The other IL-17 receptors are IL-17RB–E, each containing their own distinct IL-17 binding specificities. IL-17 receptors are broadly expressed not only by leukocytes, but also by endothelial cells and fibroblasts, amongst others (Fossiez et al., 1996). Therefore, the IL-17 family of cytokines contains several related cytokines and receptors that are generally pro-inflammatory.

IL-23 is a heterodimer composed of p19 and p40 subunits, of which the p40 subunit is shared with IL-12 (Oppmann et al., 2000). IL-23 is most prominently known as a driver of T_H_17 cell expansion and IL-17 production (Aggarwal et al., 2003). Besides a role in promoting T_H_17 cell expansion, IL-23 induces IL-1, TNF, and IL-6 from innate immune cells (Murphy et al., 2003; Uhlig et al., 2006). Importantly, IL-23 also promotes IL-17 production from _γδ_T cells and natural killer (NK) T cells (Sutton et al., 2009; Liu et al., 2011b). Therefore, IL-23 has a prominent role in promoting IL-17 production by both T_H_17 cells and innate immune cells.

### MECHANISMS GOVERNING IL-17 PRODUCTION BY LEUKOCYTES

The requirements for the generation of T_H_17 cells have been intensively studied, and depend primarily on the presence of transforming growth factor (TGF)-β and IL-6 during lymphocyte activation (for a review, see McGeachy and Cua, 2008). Also, later stages of T_H_17 cell development and maximum IL-17 production are dependent on IL-23. While these pathways have become quite well defined in recent years, the pathways responsible for IL-17 production by leukocytes of innate immune lineages remain less clear. Evidence for innate sources of IL-17 came with the observation that IL-17 was present in recombination-activating gene (RAG)-deficient mice, which lack mature B and T cells (Uhlig et al., 2006). Further evidence has accumulated showing that substantial amounts of IL-17 can be produced by _γδ_T cells, NK T cells, Paneth cells, mast cells, neutrophils, and macrophages (Lockhart et al., 2006; Michel et al., 2007; Takahashi et al., 2008; Hueber et al., 2010; Li et al., 2010; Bosmann et al., 2012). We and others have reported that activation of Toll-like receptor 4 (TLR4) on mouse peritoneal and alveolar macrophages results in the MyD88-dependent production of IL-17 and IL-17F (Gu et al., 2008; Bosmann et al., 2011, 2012). This effect appears to be TLR4-specific, because activation of other TLRs does not result in the production of these cytokines. However, one report has described the generation of IL-17 in response to TLR2 activation by chitin (Da Silva et al., 2008). The molecular mechanisms that regulate IL-17 production by innate immune cells remain otherwise undefined. Lymphocytic innate cells (e.g., NK T cells, _γδ_T cells) appear to rely on the transcription factor RORγt for IL-17 production, much like conventional T_H_17 cells (Cua and Tato, 2010). Recently, mast cells were also shown to depend on RORγt for IL-17 gene transcription (Hueber et al., 2010). Interestingly, RORγt was elevated in IL-10^–/–^ macrophages and enhanced IL-17 production (Gu et al., 2008). However, TLR4-activated wild type macrophages, which produced lower levels of IL-17, had no detectable RORγt expression (Gu et al., 2008). Therefore, the transcription factors relevant for IL-17 induction by macrophages remain unclear.

## MODULATION OF IL-17 AND IL-23 BY C5a

### MODULATION OF INNATE IL-17 AND IL-23 PRODUCTION BY C5a

The complement activation product, C5a, has been shown to modulate IL-17 production by innate immune cells. We have recently reported that C5a alters the production of both IL-17 and IL-17F, and IL-23 in LPS-stimulated macrophages. Specifically, the production of IL-17 and IL-23 by TLR4-stimulated macrophages *in vitro* was significantly reduced in a dose-dependent manner in the concomitant presence of C5a (Bosmann et al., 2012). Using genetically targeted C5aR- or C5L2-deficient mice, it was shown that the inhibitory effects of C5a on IL-17 and IL-23 production are mediated exclusively by C5aR. Mechanistically, the modulatory effects of C5a on IL-17 and IL-23 appear to be mediated indirectly by C5aR activation-induced enhancement in IL-10 release, which reduces IL-17 and IL-23 in an autocrine/paracrine manner; however, a direct mechanism was not ruled out. In agreement with these findings, C5a inhibited IL-23 release by TLR-activated human monocyte-derived dendritic cells (DCs; Zaal et al., 2012). Interestingly, while C5a down-regulated IL-17 and IL-23 production by macrophages, it has been shown to enhance the release of IL-17F. Specifically, while TLR4 activation induces production of IL-17F by macrophages *in vitro*, the co-presence of increasing concentrations of C5a results in a synergistic increase in IL-17F release (Bosmann et al., 2011). The heightened release of IL-17F is mediated through combined enhancement of the phosphatidylinositol 3-kinase (PI3K)/Akt pathway by C5a and LPS. Taken together, these studies reveal that C5a modulates IL-17, IL-17F, and IL-23 production by macrophages, which has the potential to significantly alter immune responses.

**FIGURE 1:**
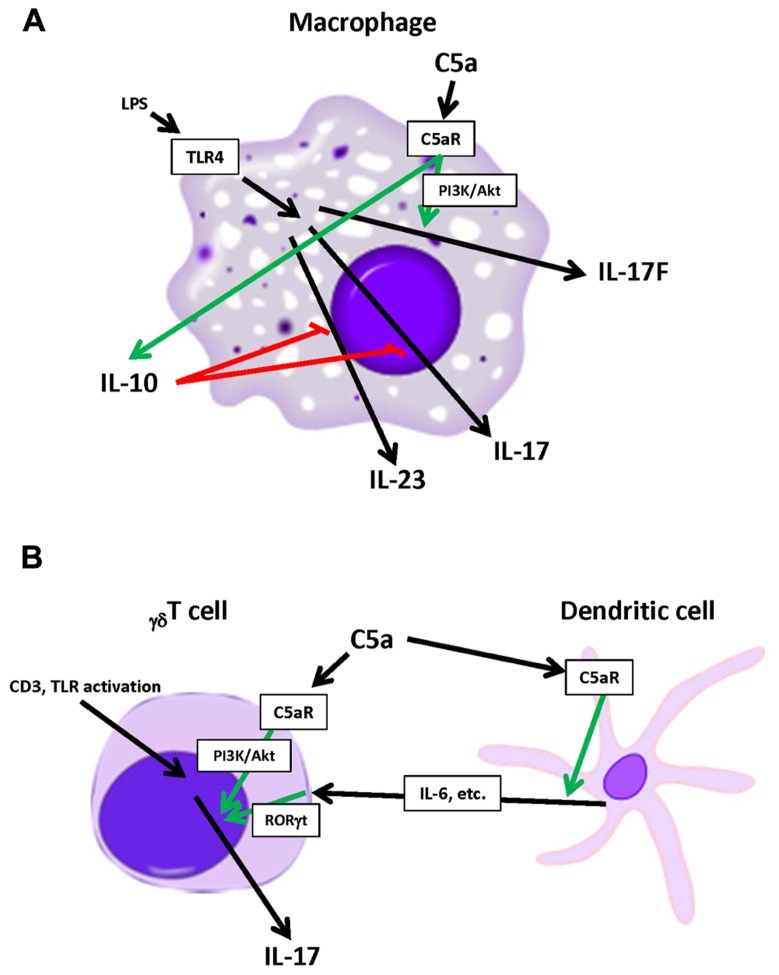
**Modulation of innate immune cell-derived IL-17 by C5a**. **(A)** LPS-activated macrophages produce IL-17, IL-17F, and IL-23. C5aR activation has been shown to enhance IL-17F while reducing IL-17 and IL-23 production by macrophages. **(B)** Activated _γδ_T cells produce IL-17 which is enhanced directly or indirectly by C5a.

Studies have shown that the production of IL-17 by _γδ_T cells can also be affected by C5a. Activation of C5aR on _γδ_T cells enhanced IL-17 production following CD3 ligation *in vitro*, through a PI3K/Akt-dependent manner (Han et al., 2011). Furthermore, IL-17 production by _γδ_T cells can be enhanced indirectly. Specifically, the activation of DCs by C5a promoted the production of IL-17 by _γδ_T cells, which appeared to be the result of enhanced IL-6 production by C5a-activated DCs (Xu et al., 2010). These results indicate that C5a significantly alters IL-17 production by cells of the innate immune system, as summarized in **Figure 1**.

In addition to modulating IL-17 production by innate leukocytes *in vitro*, C5a can modulate TLR-induced IL-17 production *in vivo*. Specifically, co-injection of C5a and Pam_3_CSK_4_ (prototypic TLR2 ligand) resulted in a synergistic enhancement in IL-17 production compared to each agonist alone when injected into the gingiva of mice (Abe et al., 2012). In the same study, administration of a C5aR antagonist resulted in reduced production of IL-17 during two models of periodontitis. Therefore, modulation of the complement system may be a potential therapeutic target for modulating (either up or down) innate immune cell-derived IL-17 production and thus innate immune responses.

### MODULATION OF IL-17 IN ACQUIRED IMMUNITY BY C5a

C5a has been shown to modulate the phenotype of T cells through indirect mechanisms, owing to the fact that C5a receptors are little, if at all, expressed on mature T cells (Dunkelberger et al., 2012), although antigen-stimulated CD4^+^ T cells may have low levels of C5aR expression (Strainic et al., 2008). Importantly, C5a alters cytokine production by DCs and tissue macrophages in a manner that can promote or inhibit the generation of T_H_17 cells. Specifically, the cytokine milieu produced by TLR2- and OVA-activated C5aR^–/–^ DCs was substantially different from wild type cells, and included increased production of TGF-β, IL-6, IL-21, and IL-23 *in vitro*, cytokines important for T_H_17 differentiation (Weaver Jr. et al., 2010). Furthermore, DC or macrophage C5aR deficiency or blockade with a C5aR antagonist promoted T_H_17 cell differentiation both *in vitro* and *in vivo* (Fang et al., 2009; Weaver Jr. et al., 2010). In agreement with these findings, another report determined that IL-17 levels in experimental asthma are reduced by signaling through C5aR (Lajoie et al., 2010). Specifically, antibody blockade or genetic disruption of C5aR or C5 enhanced T_H_17 cell differentiation following house dust mite extract challenge. In the same study, the genetic absence of C3aR reduced IL-17 production, indicating opposite effects of C5aR and C3aR. The other C5a receptor, C5L2, has also been shown to modulate IL-17 production by T cells. C5L2^–/–^ DCs released substantial amounts of IL-23 following stimulation with house dust mite allergen *in vitro*, an effect not observed in wild type DCs (Zhang et al., 2010). Furthermore, C5L2^–/–^ DCs significantly enhanced the production of T_H_17 cells and IL-17 *in vitro* and *in vivo* during experimental asthma, compared to wild type controls (Zhang et al., 2010). Taken together, these reports demonstrate that the absence of either C5aR or C5L2 on DCs enhances T_H_17 differentiation by altering the local cytokine milieu. Therefore, C5a-induced activation of DCs inhibits the generation of T_H_17 cells and IL-17 production both *in vitro* and *in vivo*.

While C5a appears to reduce lymphocyte-derived IL-17, conflicting data has been generated. Specifically, during complement-dependent experimental autoimmune arthritis, C5aR deficiency blocked the generation and expansion of T_H_17 cells, which suppressed the development of arthritis (Hashimoto et al., 2010). This effect was determined to be the consequence of modulation of local cytokine production by tissue resident macrophages to promote a T_H_17-type response. Similarly, C5a has been reported to enhance IL-17 production by human T cells *in vitro*, which is dependent on enhanced IL-6 and IL-1β expression from monocytes (Liu et al., 2011a). Therefore, C5a has also been shown to promote T_H_17 cell differentiation and enhance IL-17 production by T cells.

**FIGURE 2:**
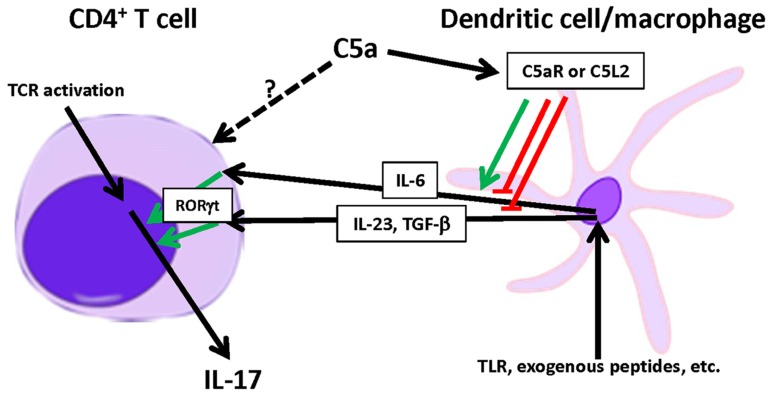
**Modulation of T cell-derived IL-17 by C5a.** C5a modulates T_H_17 cell differentiation by altering the local production of IL-23, IL-6, and TGF-β by DCs or macrophages. C5a can either enhance or inhibit T_H_17 cell differentiation depending on the inflammatory context.

Taken together, these reports demonstrate that the effect of C5a on T cell-derived IL-17 remains controversial, as summarized in **Figure 2**. It is apparent that the actions of C5a are context-dependent and may be reliant on the target cell and the co-presence of other activators or regulators. Therefore, further study is needed to delineate the mechanism of C5a-induced changes in IL-17 during acquired immune responses.

### C5a-MEDIATED MODULATION OF THE IL-17 FAMILY *IN VIVO*

#### Endotoxemia and sepsis

Evidence has accumulated that reveals a role of C5a for modulating IL-17 during immune responses *in vivo*. IL-17 has a key role during endotoxemia and sepsis, evidenced by the finding that antibody blockade of IL-17 reduces mortality following lethal LPS administration or cecal ligation and puncture (CLP; Flierl et al., 2008; Bosmann et al., 2012). Importantly, C5aR^–/–^ mice displayed significantly enhanced serum IL-17 production during endotoxemia, indicating that C5aR activation limits plasma IL-17 levels. In contrast to IL-17, IL-17F in plasma is reduced by blocking C5a during endotoxemia (Bosmann et al., 2011). These observations likely result from the finding that significant production of IL-17 during endotoxemia is from macrophages (Bosmann et al., 2012), and C5a differentially affects macrophage-derived IL-17 and IL-17F as described above (see Modulation of IL-17 in Acquired Immunity by C5a). IL-17 production during CLP is also altered by C5a. Specifically, blockade of C5a significantly reduced IL-17 in plasma, and also reduced mortality during CLP (Xu et al., 2010). In the same report, _γδ_T cells were determined to be the predominant source of IL-17. Therefore, C5a can significantly alter IL-17 levels during endotoxemia and sepsis.

#### Autoimmune and chronic inflammatory diseases

IL-17 has been shown to have a prominent role in autoimmune and chronic inflammatory diseases such as asthma, rheumatoid arthritis, and experimental autoimmune encephalomyelitis (EAE; Chabaud et al., 1999; Komiyama et al., 2006; Kudo et al., 2012). Importantly, C5a has been shown to modulate IL-17 levels in these diseases. In asthma, the production of IL-17 and airway hyper-responsiveness are regulated, in part, by C5a. A negative linear correlation between serum C5a concentration and lung T_H_17 cell numbers was observed following house dust mite extract challenge (Lajoie et al., 2010). The same report demonstrated that mice deficient in C5 produced significantly more IL-17A during experimental asthma. Antibody blockade of C5aR enhanced T_H_17 cell numbers. Furthermore, C5L2^–/–^ mice have increased pulmonary IL-17, indicating a role for the second C5a receptor *in vivo* (Zhang et al., 2010). Therefore, C5a negatively regulates IL-17 production during experimental asthma, which results in reduced airway hyper-responsiveness.

C5a also modulates IL-17 during experimental arthritis. In a complement-dependent autoimmune arthritis model, C5a activated tissue macrophages to produce IL-6 and drive T_H_17 cell differentiation (Hashimoto et al., 2010). Furthermore, C5aR deficiency resulted in significant reductions in auto-reactive T_H_17 cells and disease severity. Therefore, C5a modulates IL-17 during autoimmune arthritis. However, the role of C5a on modulating IL-17 during other arthritis models (e.g., collagen-induced) has not been demonstrated, which limits the scope of this single report.

Complement has been reported to modulate IL-17 during EAE. Specifically, the genetic ablation of decay accelerating factor (DAF), a complement regulatory protein that blocks the generation of C3a and C5a by blocking C3 convertase results in enhanced T_H_17 cell production and increased injury during EAE (Liu et al., 2008). Importantly, the absence of either C5aR or C3aR reduced T_H_17 cell differentiation (Liu et al., 2008). Furthermore, either augmenting DAF levels using over-expressing transgenic mice or use of FUT-175, the C3/C5 convertase inhibitor, reduced EAE severity and T_H_17 cell numbers (Li et al., 2009a,b). Taken together, these reports indicate that complement can enhance T_H_17 cell accumulation and disease severity during EAE. However, the exact mechanism of this modulation remains unclear.

## CONCLUSION

Since the discovery of IL-17, it has become clear that specific pathogens trigger IL-17-dependent responses and that robust IL-17 production is required for the efficient clearance of many pathogens. The mechanism responsible for this phenomenon remains unclear, but appears to be dependent on the ability of specific pathogen-associated molecular pattern (PAMP)-dependent signals to promote the production of IL-17 and/or IL-17-enhancing cytokines (e.g., IL-23) by innate immune cells. The role of complement in modulating IL-17-dependent inflammation is only now becoming apparent. Due to the fact that the effects of C5a on IL-17 vary significantly based on the inflammatory context and target cell type, making generalized conclusions about these interactions are difficult. Many questions regarding C5a-induced modulation of IL-17 remain, and the relationship between these two mediators deserves significant attention. The clinical importance of both IL-17 and C5a for a myriad of inflammatory diseases has been extensively demonstrated. Therefore, an understanding of the relationship between the two mediators has significant clinical implications for the treatment of both acute and chronic inflammatory diseases.

## Conflict of Interest Statement

The authors declare that the research was conducted in the absence of any commercial or financial relationships that could be construed as a potential conflict of interest.
